# Short-Term Administration of Parathyroid Hormone Improves Wound Healing Around Implants in an Osteoporotic Rat Model

**DOI:** 10.3390/jcm14113900

**Published:** 2025-06-01

**Authors:** Farah A. Al-Omari, Shinichiro Kuroshima, Ryohei Kozutsumi, Takashi Sawase

**Affiliations:** 1 Department of Applied Prosthodontics, Graduate School of Biomedical Sciences, Nagasaki University, Nagasaki 852-8588, Japan; omarii.farah@gmail.com (F.A.A.-O.); kozutsumi@nagasaki-u.ac.jp (R.K.); sawase@nagasaki-u.ac.jp (T.S.); 2 Division of Orthodontics, College of Dentistry, The Ohio State University, Columbus, OH 43210, USA; 3 Department of Fixed and Regenerative Prosthodontics, Division of Oral Functional Science, Faculty of Dental Medicine, Hokkaido University, Sapporo 060-0813, Japan

**Keywords:** osteoporosis, dental implant, parathyroid hormone, bone quality, tissue healing

## Abstract

**Background/Objectives**: Intermittent parathyroid hormone (PTH) administration increases bone quantity. Existing animal studies have revealed improvements in tissue healing around implants after PTH administration. It is still unclear whether PTH has a beneficial short-term effect on the early healing of bone and soft tissue around implants in individuals with osteoporosis. The current study aims to examine whether short-term intermittent PTH administration accelerates and improves early tissue healing around implants in ovariectomized rats. **Methods**: Ovariectomized rats received implants at the healed sites of extracted maxillary first molar sockets 12 weeks after the ovariectomy surgery. A daily dose of PTH was subcutaneously administered in the test group, whereas saline was administered for the control group. Long bones and maxillae were harvested 1 week after PTH administration. The following criteria were assessed: quantity and quality of long bones and peri-implant bone, bone healing around the implants, and soft tissue healing. **Results**: PTH significantly increased the bone parameters of long bones. Moreover, the bone volume around the implant increased significantly compared to controls. Improved bone quality was indicated through PTH administration by increased numbers of osteoblasts and osteoclasts, as well as decreased quantities of sclerostin + osteocytes. Furthermore, PTH administration significantly improved soft tissue healing, promoted collagen production and angiogenesis, and increased the numbers of macrophages in the connective tissue around the implants. **Conclusions**: Short-term intermittent PTH administration significantly accelerates soft tissue healing, which could lead to enhanced early osseous healing and bone formation around implants. Thus, Intermittent PTH administration might be considered as an available treatment modality for dental implants in osteoporosis patients.

## 1. Introduction

According to the recent literature, the overall prevalence of diagnosed osteoporosis in postmenopausal women was 17.4% between 2005 and 2018 [1]. Moreover, the prevalence of osteoporosis in women aged 65 years and older was four times higher than in those under 65 [1]. Compounding the problem, the United Nations estimates that the proportion of the global population aged 60 and over will nearly double (2.1 billion), increasing from 12% to 22% by 2050 [2]. Osteoporosis is the most prevalent skeletal disorder affecting bone strength, which results in an increased risk of fragility fractures [3]. Bone strength is determined by bone mineral density (BMD) and bone quality. BMD is measured in grams of mineral per area or volume of bone [3], whereas bone quality consists of bone mineralization, bone turnover, damage accumulation, and bone architecture, as defined by the National Institutes of Health in 2001 [3]. Our previous data have demonstrated that bone quality is linked to several factors, including bone cells (such as osteoblasts, osteoclasts, and osteocytes), collagen fibers, and the biological apatite c-axis [4,5,6,7].

The popularity of dental implants is increasing at an annual rate of approximately 14% [8]. According to the American Dental Association, around 5 million dental implants are placed each year in the U.S. [9]. In conjunction with this, the percentage of implant patients aged over 70 years has experienced a significant and rapid increase, rising from 7.7% to 21.0% between 2002 and 2014 [10]. Thus, the utilization of dental implant treatment among elderly patients is expected to increase. Several potential concerns with dental implant treatment in elderly patients include the risk of impaired wound healing [11], the healing period [12], and determining the optimal loading protocol [13]. The decision to opt for immediate or early loading of dental implants should be guided by clinical parameters, bone density, and the primary stability of the implants [13]. Consequently, significant peri-implant bone loss has been observed in osteoporotic patients, with implant failure rates as high as 4.8% [14]. Thus, an acceleration and improvement of tissue healing, as well as maintaining the peri-implant bone around the dental implant, should be clinically required in the elderly population.

Parathyroid hormone (PTH) is an FDA-regulated anabolic agent to reduce the risk of bone fracture in osteoporosis patients [15,16]. PTH regulates bone tissue remodeling by controlling serum calcium and phosphate levels [17], in addition to controlling different bone cells [18,19,20]. Several animal studies have shown that intermittent administration of PTH stimulates bone production by increasing the number of osteoblasts [18] and reducing the *Sost* gene expression in osteocytes [19]. Furthermore, intermittent and continuous PTH administration increases the number of osteoclasts by elevating RANKL mRNA levels (*TNFSF11*) and decreasing osteoprotegerin (OPG) gene (*TNFRSF11B*) expression, resulting in increased osteoclastogenesis in rats [20]. Also, alterations in cortical bone mineral content and structural properties can be observed in mice following 2–3 weeks of PTH treatment [21]. This approach showed significant localized changes in murine bone mineral content in the medial and posterior regions of the proximal tibia after one week of PTH administration [22]. PTH may also aid in the healing of soft tissues by reducing inflammation and promoting collagen deposition [5,6,23]. The caveat here is that continuous exposure to PTH may exacerbate bone loss, and the development of hypercalcemia remains a significant concern, potentially resulting in clinical manifestations such as nephrolithiasis, nausea, and gastrointestinal disturbances [16]. In preclinical models, elevated PTH levels—whether experimentally induced or tumor-associated—can lead to symptoms including vomiting, constipation, and lethargy. Furthermore, long-term PTH administration may necessitate subsequent antiresorptive therapy, as rapid bone loss has been observed following treatment cessation [24].

Prior research from our group showed that administering PTH intermittently via the intraoral route promoted faster healing of both bone and soft tissues in rat extraction sockets [24]. Additionally, 5-week subcutaneous intermittent administration of PTH significantly increased bone volume and bone mineral density (BMD) and improved the bone quality around implants placed in rat tibiae [12]. In another animal study, the data showed that intermittent PTH administration improves medullary bone-to-implant contact in adult rats’ tibiae [25]. Despite existing research, the role of short-term systemic intermittent subcutaneous PTH in promoting peri-implant tissue healing in osteoporotic individuals is yet to be determined. The objective of this study is to assess whether short-term intermittent administration of PTH confers a measurable biological advantage in promoting early bone and soft tissue regeneration around implants placed in osteoporotic rat maxillae.

## 2. Materials and Methods

### 2.1. Animals, Implant Designs, Surgical Procedures, and Intermittent PTH Administration

Ten ovariectomized Wistar rats at 7 weeks of age were obtained (Kyudo Co., Ltd., Saga, Japan) to receive sandblasted, acid-etched, and grade IV titanium implants (Kyocera Co. Ltd., Kyoto, Japan). The implants were 2.0 mm in diameter and 3.5 mm in length. Both maxillary first molars were carefully extracted at week 16, without causing root fractures, nine weeks after ovariectomy. Implants were placed in the healed extraction sites three weeks post-extraction. A combination of ketamine (90 mg/kg) and xylazine (10 mg/kg) was used to induce general anesthesia for all procedures (Daiichi-Sankyo and Elanco Japan Co., Ltd., respectively; Tokyo, Japan). To induce estrogen-deficiency-like osteoporosis, we waited for a period of 12 weeks between the ovariectomy and implant placement [26]. Postoperative care included intraoral observation and irrigation with sterile saline every three days to avoid infection of the wounds. Two rats were housed per cage, with free access to water and food, in a controlled environment with a 12/12 light/dark cycle. The animal care and experimental procedures adhered to the Guidelines for Animal Experimentation of Nagasaki University and were approved by the Ethics Committee for Animal Research (Approval number: 160809133-1-4; approval date: 30 July 2021). The experiments were reported according to the ARRIVE guidelines (https://arriveguidelines.org/arrive-guidelines, accessed on 10 October 2024).

**Figure 1:**
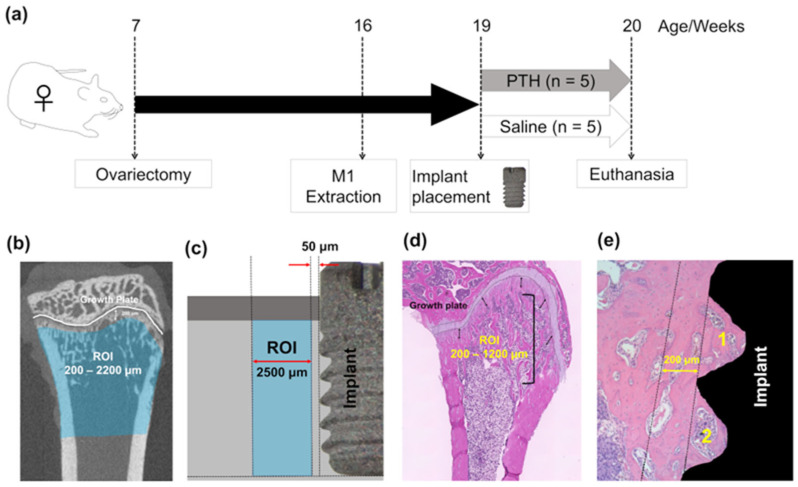
Experimental design: (**a**) Experimental timeline. Ovariectomized female rats received implants in the extracted maxillary first molar socket 12 weeks after surgery. Rats were randomly divided into 2 groups (n = 5/each group). (**b**) Microcomputed tomography (microCT) analysis for the rats’ tibiae. Regions of interest (ROIs) were determined between 200 and 2200 µm away from the tibial metaphysis. (**c**) Areas of interest (AOIs) for the femoral histomorphometric analyses were determined between 200 and 1200 µm away from the growth plate. (**d**) MicroCT analysis identified ROIs within a range of 50–2500 μm from the implant surface in the maxilla. (**e**) AOIs for histomorphometric and immunohistochemical analyses were identified and examined separately inside and outside up to 200 μm from the first and second threads of the implant in the rats’ maxillae. The 1 and 2 indicate the first and second threads, respectively.

The rats were randomly divided into two groups on the day of implant placement surgery, with five rats per group. Intermittent subcutaneous injection of human PTH (PTH [1,2,3,4,5,6,7,8,9,10,11,12,13,14,15,16,17,18,19,20,21,22,23,24,25,26,27,28,29,30,31,32,33,34]; BACHEM, Bubendorf, Switzerland) was given to one group via the dorsal neck region (PTH group), while the control group received saline injections. The PTH was injected daily in the morning at a dose of 80 μg/kg, and with a volume between 120 and 140 μL, for one week [5,6,24]. Carbon dioxide gas inhalation was used at the end of the injection period for euthanasia, after which the tibia, femur, and maxillae with the inserted implants were collected (Figure 1a). All implants successfully achieved osseointegration without any incidents, such as implant disintegration or death during or after the procedures, including tooth extraction, implant placement, and PTH administration. Thus, no rats were excluded at the time of euthanasia. Implant osseointegration was assessed by gently probing and removing the implants using forceps. All experimental procedures—extractions, implant insertions, and PTH treatments—were conducted by a single operator (F.A.A.-O.) to maintain consistency. Moreover, all histological analyses were performed blindly by the results assessor (F.A.A.-O.), with the sample labeling masked to avoid potential biases during analysis.

### 2.2. Assessment of Long Bone

Dissected tibiae and femora were fixed in 10% neutral buffered formalin (Muto Pure Chemicals Co., Ltd., Tokyo, Japan) for 48 h at 4 °C (n = 5 per group). MicroCT scans were performed on the tibiae at a 20 μm voxel resolution with an energy level of 90 kV (R_mCT2; Rigaku Co., Tokyo, Japan). Regions of interest (ROIs) were defined between 200 μm and 2200 μm from the proximal tibiae along the longitudinal axis to exclude any influence from the implants (Figure 1b). To assess the effects of PTH on long bones, measurements were taken for trabecular bone volume per tissue volume (Tb.BV/TV), trabecular number (Tb.N), trabecular thickness (Tb.Th), trabecular separation (Tb.Sp), trabecular bone mineral density (Tb.BMD), cortical bone ratio (cortical bone volume per total bone volume: Cv/Av), cortical bone thickness (Ct.Th), and the external and internal perimeters of cortical bone (external and internal line lengths, respectively). These measurements were conducted semi-automatically using TRI/3D-Bon (Ratoc System Engineering, Tokyo, Japan), following established guidelines for microCT analysis [27].

The dissected femora were fixed in 10% neutral buffered formalin for 48 h at 4 °C and subsequently demineralized in ethylenediaminetetraacetic acid (pH 7.3, FUJIFILM Wako Pure Chemical Co., Osaka, Japan) at 4 °C for 42 days (n = 5 per group). The demineralized femora were then embedded in paraffin and sectioned into 5 μm thick slices using a microtome (REM-710 Litratome, Yamato Kohki Industrial Co., Ltd., Saitama, Japan). The sections were stained for histomorphometric analysis using hematoxylin and eosin (H&E) (FUJIFILM Wako Pure Chemical Co.) and tartrate-resistant acid phosphatase (TRAP) (386 A; Sigma–Aldrich, St. Louis, MO, USA), following the manufacturer’s protocols. Areas of interest (AOIs) were defined between 200 μm and 1200 μm from the growth plate (Figure 1c). The stained sections were examined and photographed under light microscopy (Axio Scope A1; Zeiss, Oberkochen, Germany). In these AOIs, trabecular and cortical bone area fractions (Tb.BA/TA and Ct.BA/TA, respectively) and osteoclast numbers per bone perimeter (N.Oc/BS) were assessed.

### 2.3. Bone Quantity Assessment Around the Implant

Samples consisting of maxillary bone and implants were trimmed and fixed in 10% formalin at 4 °C for 48 h (n = 5). The samples were then analyzed using microCT and H&E staining to evaluate the bone quantity around the implants. MicroCT scanning was performed at a voxel resolution of 20 μm and a tube voltage of 90 kV (R_mCT2; Rigaku Co.). Semi-manual measurements were used to segment and reconstruct the bone surrounding the implant (Ratoc System Engineering). A range of bone parameters were semi-automatically calculated using TRI/3D-Bon software, following guidelines for bone structure analysis with microCT [27]. To reduce the impact of metal artifacts, the analysis was limited to a horizontal distance of 50 μm to 2500 μm from the implant surface, as artifacts typically occur within 50 μm around the implants (Figure 1d) [28].

After microCT scanning, the maxillary blocks were decalcified in 10% ethylenediaminetetraacetic acid for 42 days at 4 °C. Following decalcification, the implants were carefully removed by manual counterclockwise rotation. The maxillary blocks were then embedded in paraffin and sectioned into 5 μm thick slices using a microtome. The bone area fraction (BA/TA) was assessed in H&E-stained sections within two separate areas of interest (AOIs): (1) between the first and second threads of the implant, and (2) in the surrounding tissue extending up to 200 μm from the outer edge of the threads (Figure 1e)

### 2.4. Assessment of Bone Quality Around the Implant

The bone quality around the implants was evaluated through staining of bone cells, including osteocytes, osteoblasts, and osteoclasts, alongside histomorphometric analysis [4,6,7]. Semi-automated quantitative analysis was conducted using NIH ImageJ (version 1.8.0_172; National Institutes of Health, Bethesda, MD, USA). The number and density of osteocytes within the AOIs were assessed from H&E-stained sections. TRAP staining was used to identify multinucleated osteoclasts, and N.Oc/BS was determined accordingly.

Immunohistochemical staining was employed to identify osteoblasts and osteocytes. Paraffin-embedded sections were incubated overnight at 4 °C with primary antibodies: rabbit polyclonal anti-Runx2 at dilution of 1:800 (ab23981; Abcam, Cambridge, MA, USA) for osteoblasts, and goat anti-mouse sclerostin (1:25 dilution, AF1589; R&D Systems, Minneapolis, MN, USA) for osteocytes. Detection was performed using HRP-conjugated secondary antibodies: goat anti-rabbit IgG (1:1000 dilution; ab6721, Abcam) and rabbit anti-goat IgG (1:1000 dilution; SA0001-4, Proteintech, Rosemont, IL, USA). Protein expression was visualized using the DAB Substrate Kit (ab64238; Abcam) and counterstained with methyl green (Wako Pure Chemical Industries, Ltd., Richmond, VA, USA). The density of Runx2-positive osteoblasts and sclerostin-positive osteocytes was calculated semi-automatically.

### 2.5. Soft Tissue Healing Assessment Around the Implant

Healing was assessed by staining the connective tissue and blood vessels around the implants. The areas of interest (AOIs) were delineated as spanning 2000 μm horizontally from the implant surface and vertically from the crestal bone crest to the terminal edge of the connective tissue. Masson’s trichrome staining (HT15; Sigma-Aldrich) was performed in accordance with the manufacturer’s protocol to visualize collagen fibers and assess collagen production.

Immunohistochemical staining was performed to identify blood vessels within the soft tissue. Sections were dehydrated, blocked, and then incubated overnight at 4 °C with a rabbit polyclonal antibody against von Willebrand factor (vWF) (1:800 dilution, ab6994; Abcam). Sections were incubated with 1:1000 dilution secondary antibody (ab6721, Abcam), followed by DAB development and counterstaining. The density of vWF-positive blood vessels was then investigated and quantified.

To examine macrophage distribution and density in the connective tissue around the implants, immunofluorescent staining was performed. Sections underwent trypsin antigen retrieval (ab970, Abcam) and blocking before being incubated overnight at 4 °C with mouse monoclonal anti-CD68 (1:100 dilution, ab3163; Abcam), a marker for pan-macrophages. Alexa Fluor 546 donkey anti-mouse IgG was used as the secondary antibody, with dilution of 1:200 (A10036; Invitrogen, Carlsbad, CA, USA). For final mounting of the sections, a mounting medium with DAPI (H-1800; VECTASHIELD Vibrance Antifade; Vector Laboratories, Burlingame, CA, USA) was used for final mounting of the sections. CD68-positive macrophages were counted, and their density was calculated.

### 2.6. Statistical Analysis

All statistical analyses were conducted with blinding to ensure unbiased evaluation. Normality was assessed using the Shapiro–Wilk test. The independent *t*-test was employed to compare the control and PTH groups. Significance was set at a *p*-value < 0.05, consistent with our previous animal study [6]. Systat 13 (Systat Software) was utilized for statistical analyses. Means ± SE represented the data.

## 3. Results

### 3.1. The Effect of PTH on Long Bone

**Figure 2:**
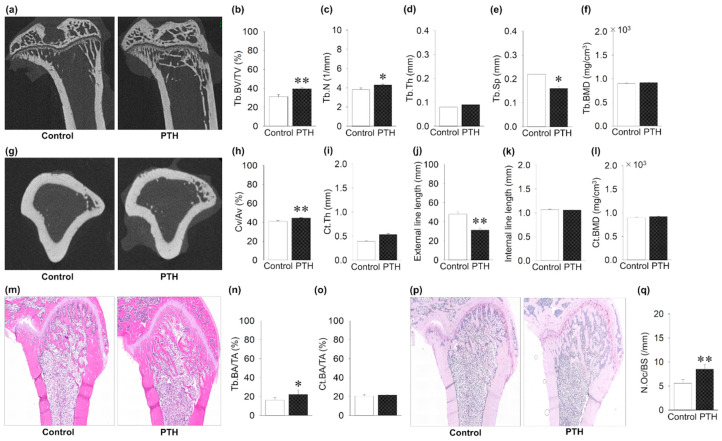
Effects of intermittent parathyroid hormone (PTH) administration on tibial long bone: (**a**) Representative microcomputed tomography (microCT) images of the tibial metaphysis. (**b**,**c**) Trabecular bone volume per tissue volume (Tb.BV/TV) and trabecular number (Tb.N) are significantly increased in the PTH group compared to the control group. (**d**) Trabecular thickness (Tb.Th) is the same between the groups. (**e**) Trabecular separation is significantly decreased in the PTH group compared to the control group. (**f**) Trabecular bone mineral density (Tb.BMD) is the same between the PTH and control groups. (**g**) Representative images of microCT-constructed cross-sectional images. (**h**) Cortical volume ratio (Cv/Av) is significantly increased in the PTH group compared to the control group. (**i**) Cortical thickness (Ct.Th) is the same between groups. (**j**) External length of cortical bone is significantly decreased in PTH compared to controls. (**k**) Internal length of cortical bone is almost the same between groups. (**l**) Cortical bone mineral density (Ct.BMD) is the same between the PTH and control groups. (**m**) Representative hematoxylin- and eosin-stained images. (**n**) Trabecular bone surface area (Tb.BA/TA) shows a significant increase in PTH compared to the control group. (**o**) Cortical bone surface area (Ct.BA/TA) is the same between groups. (**p**) Representative images of TRAP-stained tibial images. (**q**) Osteoclast number (N.Oc/BS) is significantly increased in PTH compared to the control group. Graphs show means ± SE; * *p* < 0.05, ** *p* < 0.01; n = 5 rats/group.

First, the effect of PTH administration on long bones was investigated to validate the action of PTH dosage and injection route. From the representative microCT images, the tibial metaphysis showed trabecular and cortical bone gain (Figure 2a,g). Intermittent PTH administration significantly increased Tb.BV/TV, Tb.N, and Cv/Av compared to the control group (Figure 2b,c,h). Intermittent PTH administration significant decreased Tb.Sp and the cortical external line length compared to the control group (Figure 2e,j). There was no effect of PTH administration on the Tb.Th, Ct.Th, and BMD. The H&E-stained paraffin sections of the femur showed a significant increase in the trabecular bone area, but not in the cortical area, in the PTH group (Figure 2m–o). Finally, intermittent administration of PTH significantly increased the osteoclast numbers as well as the TRAP staining of the femur (Figure 2p,q).

### 3.2. The Effect of PTH on Bone Quantity Around the Implant

**Figure 3:**
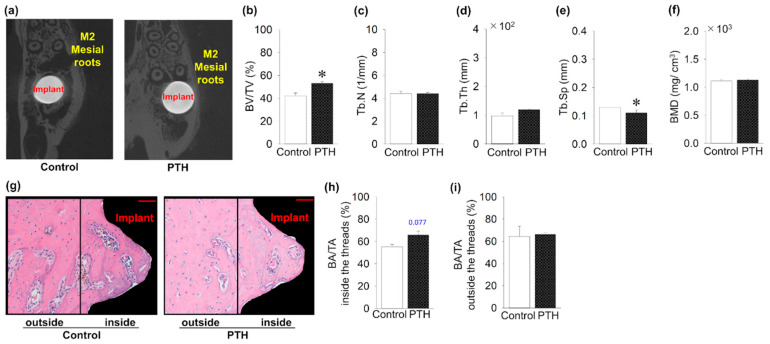
The impact of intermittent parathyroid hormone administration on bone quantity surrounding the implants: (**a**) MicroCT cross-sectional images around the implants (M2: second molar). (**b**–**f**) Bone volume fraction (BV/TV) around the implants is significantly increased in PTH compared to the control group, whereas Tb.Sp is significantly decreased in the PTH group. However, Tb.N, trabecular thickness (Tb.Th), and bone mineral density (BMD) are almost the same between groups. (**g**) Representative sagittal hematoxylin- and eosin-stained histology images around the implants (Bar = 50 μm). (**h**) Bone area fraction (BAF) showed an increase in PTH compared to the control group inside the areas of the implant threads, but it was not significant. (**i**) No significant increase in BAF was found in the areas outside the threads. n = 5 per group. Graphs show means ± SE; * *p* <  0.05.

The microCT images show the implants with the surrounding bone (Figure 3a). Intermittent administration of PTH significantly increased BV/TV compared to the control group (Figure 3b). Intermittent PTH administration significantly decreased Tb.Sp compared to the control group (Figure 3e). However, PTH administration did not affect the evaluation parameters, such as Tb.N, Tb.Th, and the BMD, as with the control group (Figure 3c,d,f). In contrast to this, the H&E sections showed an increase in the BA/TA inside the area of the implant threads in the PTH group, without significant difference (Figure 3g,h). Moreover, no significant difference in the BA/TA outside the implant thread was found (Figure 3i).

### 3.3. PTH Administration’s Effect on Bone Quality Around the Implant

**Figure 4:**
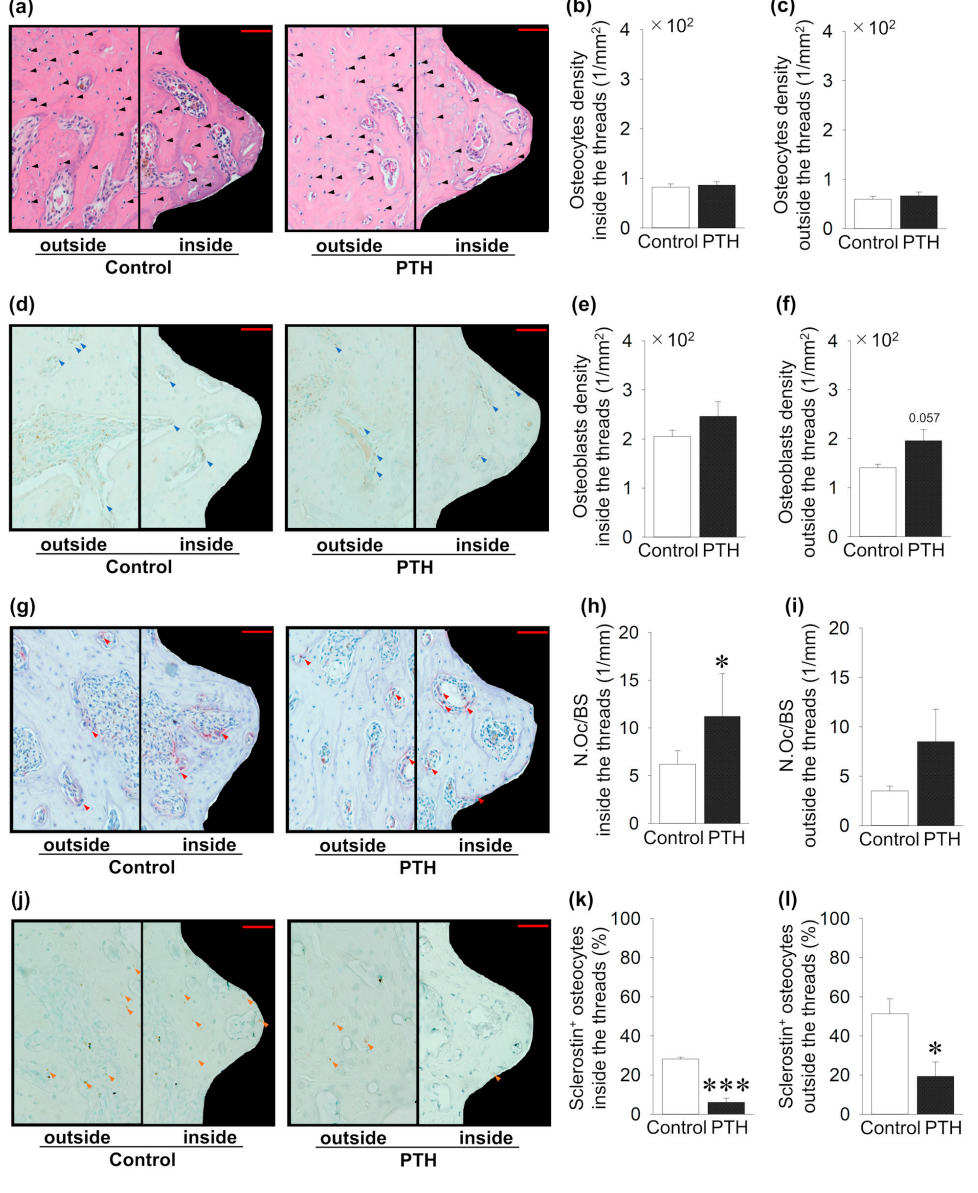
Effects of intermittent parathyroid hormone (PTH) administration on bone cells inside and outside the threads of the implants: (**a**) Representative sagittal hematoxylin- and eosin-stained images around the implants (black arrow; osteocyte). (**b**,**c**) Osteocyte density shows no change between the PTH and control groups. (**d**) Representative sagittal Runx2-immunostained images (blue arrow; osteoblast). (**e**,**f**) Osteoblast density shows no change between PTH and the control group. (**g**) Representative sagittal TRAP-stained images (red arrow; osteoclast). (**h**,**i**) The osteoclast surface (N.Oc/BS) is significantly increased in PTH compared to the control group inside the thread, whereas it is not altered outside the thread. (**j**) Representative sclerostin-immunostained images (black arrow: osteocyte; orange arrow: sclerostin + osteocyte). (**k**,**l**) Sclerostin + osteocytes are significantly decreased in PTH compared to the control group. Bar = 50 μm; n = 5 per group. Graphs show means ± SE; * *p* < 0.05, *** *p* < 0.001.

Bone cells inside and outside the implant thread were investigated, and several quantitative distributions and activity analyses were performed (Figure 4). Intermittent administration of PTH did not change the osteocyte density inside and outside the area of the threads compared to the control group (Figure 4a–c). In contrast, the density of Runx2 + osteoblasts showed an increase inside and outside the thread in the PTH group compared to the control, but it was not significant (Figure 4d–f). Intermittent administration of PTH significantly increased the number of osteoclasts per surface inside the implant thread in the PTH group, whereas PTH administration did not alter the osteoclast numbers outside the thread area (Figure 4g–i). Moreover, inside and outside the implant thread, intermittent administration of PTH showed a significant decrease in sclerostin + osteocytes ratio compared to the control group (Figure 4j–l).

### 3.4. PTH Administration’s Effect on Soft Tissue Healing Around the Implant

**Figure 5:**
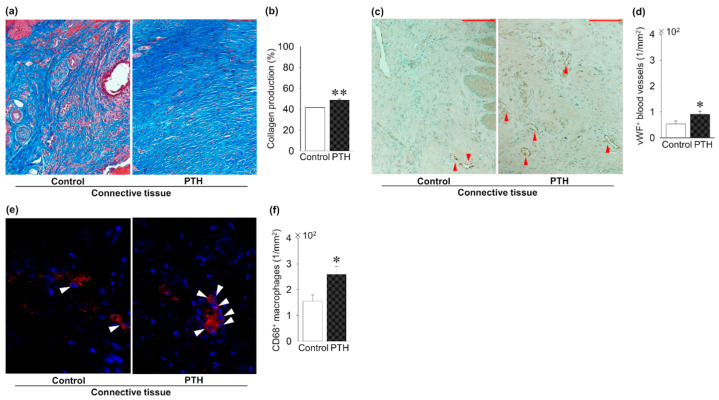
PTH administration’s effect on soft tissue regeneration around the implants: (**a**) Trichrome-stained images. (**b**) Significantly increased collagen production in PTH compared to the control group. (**c**) Representative von Willebrand factor (vWF)-immunostained images (red arrow; vWF + blood vessels). (**d**) The density of vWF + blood vessels is significantly increased in PTH compared to the control group. (**e**) Representative CD86 immunofluorescent-stained images (white arrow: CD86 + macrophages). (**f**) CD86 + macrophage density is significantly increased in PTH compared to the control group. Bar: 100 μm; n = 5 per group. Graphs show means ± SE; * *p* <  0.05, ** *p*< 0.01.

Figure 5 shows the effect of PTH administration on connective tissue around the implant. Intermittent PTH administration significantly increased the collagen production ratio in the connective tissue around the implants compared to the control group (Figure 5a,b). Intermittent administration of PTH significantly increased the density of vWF + blood vessels when compared to the control group (Figure 5c,d). Finally, intermittent administration of PTH significantly increased the CD68 + macrophage density in the connective tissue compared to the control group (Figure 5e,f).

## 4. Discussion

This study investigated the effects of short-duration intermittent systemic subcutaneous PTH administration on the early stages of osseous and soft tissue healing around the implants in ovariectomized rats’ maxillae. The results demonstrated that PTH administration for one week positively increased the bone quantity and quality around the implant by increasing the number of osteoblasts and osteoclasts inside or outside the threads and decreasing the number of sclerostin-positive osteocytes inside and outside the threads. In particular, PTH administration improved soft tissue healing around the implants by increasing the production of collagen fibers and the distribution of blood vessels and pan-macrophages in the connective tissue around the implants.

The current study employed a 12-week period between ovariectomy and tooth extraction, and a subsequent 3-week period between tooth extraction and implant insertion. It typically takes 12 weeks for ovariectomized rats to have changes in the cortical bone width of their tibial and femoral shafts [26]. Moreover, about 3 weeks is required for thick trabecular bone to form and fill the dental alveolus following tooth extraction in rats [29]. Previous studies have demonstrated that daily administration of 80 μg/kg human PTH (1–34) for 10 weeks promotes bone apposition in the mandible and alveolar crest of ovariectomized rats [30,31]. Our previous studies indicated improved healing of tooth extraction sockets in mice [24] and tissue healing around the implant in OVX rat maxillae [6] with 80 μg/kg of daily intermittent PTH administration for 2 weeks. Therefore, the abovementioned dosage and frequency, with a shorter duration, were applied in this study, although the dosage was higher than that in humans [32].

The intermittent administration of PTH has been shown to improve the radiographic and histological bone parameters of intact long bones [12,32]. In the current study, both bone quantity parameters (including trabecular bone volume, cortical bone volume, and bone area) and bone quality, indicated by the osteoclast activity, increased significantly. This is consistent with the clinical subcutaneous daily administration of PTH to treat osteoporosis patients and reduce fracture risk [15]. Previous investigations have demonstrated a significant increase in the long bone quantity and quality parameters with PTH administration [12,33,34], although the administration regimen, including the dosage, frequency, and duration, was different. In the current investigation, 80 µg/kg of human PTH (1–34) was subcutaneously administered to the rats every day in the morning period for one week. In addition, one investigation administered the PTH every second day for 5 weeks [12]. Another study administered the PTH daily for 6 weeks [34]. Regardless, the present regimen showed significant anabolic outcomes on the long bone over the short duration of PTH administration.

Several studies have investigated the effects of systemic PTH administration on improving and accelerating the formation of new bone around implants [6,12,25,35]. The current data showed a tendency for a higher bone formation around the implant under the influence of PTH administration, although the increase in bone area in the histological sections was not significant. Park et al. reported similar results of greater bone volume found in the PTH group, yet the statistical analysis showed no significant results [35]. A possible explanation is the short duration utilized in the present study, although the microCT data showed an increased bone volume around the implant in the PTH group. Adapting different dimensions for the ROI and AOI might account for the discrepancy in the results. Intermittent PTH treatment exhibits a modeling and remodeling anabolic effect, which increases the difference between the early bone formation and the later pre-existing bone remodeling [16]. Osseointegration is a different type of bone healing after bone injury following tooth extraction. Bone formation around dental implants has been demonstrated to consist of contact osteogenesis and distance osteogenesis. Contact osteogenesis is defined as bone formation on the implant surface, whereas distance osteogenesis is defined as bone growth from the marginal area of native bone [36]. Intermittent administration of PTH has been demonstrated to accelerate bone healing after tooth extraction [24], which strongly suggests that distance osteogenesis from native bone was improved after implant placement through PTH administration. PTH administration may be associated with the improvement of contact osteogenesis due to the increased number of osteoblasts with tendency and osteoclasts, as well as the decreased number of osteocytes in the current study. Therefore, short-term PTH administration may contribute to the improvement of both contact and distance osteogenesis, in terms of bone quantity.

The effects of PTH administration on bone cells vary widely, depending on dosage and duration [16]. Bone quality around implants has been investigated using the histomorphometric and immunohistochemical parameters for osteoblasts, osteoclasts, and osteocytes as indicators [6,37]. Intermittent PTH treatment directly influences osteoblasts by promoting osteoblastogenesis, reducing apoptosis, and reactivating dormant bone lining cells [16,18]. Additionally, it stimulates the expression of genes associated with bone formation, such as *Runx2*, a key transcription factor specific to osteoblasts [20]. Although the number of RUNX2 + osteoblasts in the area around the implant was increased in the present study, the difference was not significant. This might explain the lack of significant increase in the area of interest close to the implant, although microCT data showed an increase in the bone volume. Furthermore, intermittent PTH causes downregulation of *Sost* mRNA by the activation of the PTH receptor (PTH1R) on osteocytes, which subsequently promote bone formation as a result of the suppression of sclerostin production [19]. Conversely, PTH regulates osteoclastogenesis through the OPG-RANKL-RANK pathway, promoting the differentiation and survival of hematopoietic cells into fully activated osteoclasts. This leads to an increased number of osteoclasts and enhanced remodeling capacity [20], as confirmed by the current findings. Ultimately, the short duration of the intermittent PTH exposure improved the bone quality, with higher osteogenic activity regardless of the significant net effect on bone mass.

Intermittent PTH administration has been shown to enhance collagen fiber formation in the connective tissue, resulting in improved soft tissue wound healing around the implant in the present study. Our previous study also showed superior collagen fiber formation in tooth extraction sockets with intermittent PTH administration [24]. Intermittent PTH may improve wound healing by stimulating fibroblast proliferation and migration, although the underlying mechanism has yet to be fully elucidated [38]. Additionally, this treatment has demonstrated the ability to induce angiogenesis and modulate the vascularity of small-diameter blood vessels through endothelial cell PTH receptors [39,40]. Intermittent PTH also stimulates the proliferation and migration of endothelial cells [38] and recruits myeloid cells by inducing the production of interleukin (IL)-6 and chemokine (CCL)-2 from osteoblasts [41,42]. This leads to an increase in macrophage numbers and activity, which initiates phagocytosis and efferocytosis, crucial for eliminating pathogens and facilitating tissue repair [43]. Therefore, the previous data and investigations support the current findings regarding the effects of intermittent PTH administration on improved soft tissue healing around implants, including enhanced collagen fiber production and increased numbers of blood vessels and macrophages. Although the observed increase in collagen deposition and vascularization is noteworthy, it remains unclear whether these changes represent direct effects of PTH on soft tissue healing or are secondary responses resulting from enhanced bone remodeling. Further investigation is needed to delineate the underlying mechanisms driving these results.

Healing following titanium implant placement is a lengthy process that takes several months to create the marginal soft tissue attachment and the proper osseointegration [44]. Marginal soft tissue adaptation is crucial for creating a physical seal between the oral environment and the peri-implant bone. This seal helps prevent microbial organisms and contaminants from the oral cavity from reaching the underlying bone, thereby protecting it from potential infections and complications [45,46]. Such a stable peri-implant soft tissue attachment would enhance bone formation and accelerate osseointegration [44]. This is crucial for early load treatment planning decisions and ensures a long-term aesthetic outcome of implant therapy. In the current investigation, soft tissue healing showed a significant fast improvement with intermittent PTH administration. Accelerating soft tissue healing with wound closure and abundant distribution of collagen and blood vessels in the connective tissue around the implants by PTH administration could contribute to increased bone quantity and improved bone quality earlier after implant placement.

To the best of the authors’ knowledge, this study is the first to report on the impact of short-term systemic subcutaneous intermittent PTH administration on early osseous integration, bone quality, and soft tissue regeneration surrounding implants in ovariectomized rat maxillae. In a prior study, we demonstrated that intermittent PTH administration over 2 weeks was effective in improving the healing around the implants in the maxillae [6]. Interestingly, 1 week of systemic intermittent PTH administration in the current study showed comparatively significant outcomes.

Several limitations should be acknowledged when interpreting the current findings, including the relatively short duration of PTH administration, the lack of a non-osteoporotic baseline group, and the limited translational applicability inherent to this preclinical model. Additionally, this study’s scope does not account for long-term outcomes, which may be critical to fully understanding the therapeutic potential of PTH in clinical settings. Nevertheless, adapting the current animal study results into a human clinical trial will require several modifications, since the daily injection of PTH might cause patients discomfort. Moreover, achieving the optimal dosage, duration, and proper onset of PTH administration requires further investigations, as well as creating suitable patient selection criteria.

## 5. Conclusions

This study was designed to assess the impact of short-term intermittent PTH treatment on early peri-implant bone and soft tissue regeneration in a rat model of osteoporosis. While recognizing the limitations of the present work, short-term intermittent PTH administration had a positive effect on the bone formation and bone quality around the implants. Most interestingly, soft tissue healing showed a significant enhancement in the PTH group compared to the control group. The current findings suggest that short-term intermittent PTH administration may have a future consideration for a therapeutic approach to possibly enhance early tissue regeneration around implants in osteoporotic individuals.

## Data Availability

The data that support the findings of this study are available from the corresponding author upon reasonable request.
